# Clinical applicability of an artificial intelligence prediction algorithm for early prediction of non-persistent atrial fibrillation

**DOI:** 10.3389/fcvm.2023.1168054

**Published:** 2023-09-13

**Authors:** Yeji Kim, Gihun Joo, Bo-Kyung Jeon, Dong-Hyeok Kim, Tae Young Shin, Hyeonseung Im, Junbeom Park

**Affiliations:** ^1^ Cardiovascular Center, Department of Internal Medicine, College of Medicine, Ewha Womans University Medical Center, Seoul, Republic of Korea; ^2^ Interdisciplinary Graduate Program in Medical Bigdata Convergence, Kangwon National University, Chuncheon, Republic of Korea; ^3^ Department of Urology, College of Medicine, Ewha Womans University Medical Center, SYNERGY AI, Ewha Womans University Mokdong Hospital, Seoul, Republic of Korea; ^4^ Department of Computer Science and Engineering, Kangwon National University, Chuncheon, Republic of Korea

**Keywords:** non-persistent atrial fibrillation, artificial intelligence, convolutional neural network, electrocardiogram, normal sinus rhythm

## Abstract

**Background and aims:**

It is difficult to document atrial fibrillation (AF) on ECG in patients with non-persistent atrial fibrillation (non-PeAF). There is limited understanding of whether an AI prediction algorithm could predict the occurrence of non-PeAF from the information of normal sinus rhythm (SR) of a 12-lead ECG. This study aimed to derive a precise predictive AI model for screening non-PeAF using SR ECG within 4 weeks.

**Methods:**

This retrospective cohort study included patients aged 18 to 99 with SR ECG on 12-lead standard ECG (10 seconds) in Ewha Womans University Medical Center for 3 years. Data were preprocessed into three window periods (which are defined with the duration from SR to non-PeAF detection) – 1 week, 2 weeks, and 4 weeks from the AF detection prospectively. For experiments, we adopted a Residual Neural Network model based on 1D-CNN proposed in a previous study. We used 7,595 SR ECGs (extracted from 215,875 ECGs) with window periods of 1 week, 2 weeks, and 4 weeks for analysis.

**Results:**

The prediction algorithm showed an AUC of 0.862 and an F1-score of 0.84 in the 1:4 matched group of a 1-week window period. For the 1:4 matched group of a 2-week window period, it showed an AUC of 0.864 and an F1-score of 0.85. Finally, for the 1:4 matched group of a 4-week window period, it showed an AUC of 0.842 and an F1-score of 0.83.

**Conclusion:**

The AI prediction algorithm showed the possibility of risk stratification for early detection of non-PeAF. Moreover, this study showed that a short window period is also sufficient to detect non-PeAF.

## 1. Introduction

The recent Early Treatment of Atrial Fibrillation for Stroke Prevention Trial (EAST-AFNET 4 trial) showed that, compared to standard control, early rhythm control is beneficial in the general population (1). Similarly, the Risk and Benefits of Early Rhythm Control in Patients With Acute Strokes and Atrial Fibrillation trial (RAFAS trial) proved that early rhythm control may decrease the risk of recurrent stroke in acute stroke patients (2). Compared to those who received standard care, death from cardiovascular causes, stroke, or hospitalization with worsening of heart failure or acute coronary syndrome occurred less frequently in patients who received early rhythm control (3). A meta-analysis showed that early rhythm control in paroxysmal atrial fibrillation (PAF) patients with heart failure and preserved ejection fraction decreased the mortality rate (4). Thus, early rhythm control of AF has various clinical implications, but early detection of PAF is impaired by clinical limitations. Up to 30% of AF patients are asymptomatic, and the ECG-based diagnostic approach is difficult, and it becomes even more challenging to conduct an ECG for early detection of PAF at the time when symptoms appear (5–7). Attia et al. developed an artificial intelligence (AI)-enabled ECG algorithm that enables the prediction of AF during sinus rhythm and had a higher rate of diagnosis in the sinus rhythm ECG, which is taken at the time close to the occurrence of AF (8). According to the mSTOPs trial, a JAMA study, a continuous ECG patch for 2 weeks increased the rate of diagnosis of AF and especially during the first 2 weeks of symptom-onset than at 2 months later (9). BEAGLE trial, a prospective study, to evaluate the effectiveness of the model has been recently published, and it has demonstrated that risk stratification can be performed in population of older adults at risk of stroke (10). However, it still requires a monitoring period of more than two weeks and it has a limitation that clinical effectiveness has proven itself only on a specific uniform population. The anatomical remodeling and fibrosis of the left atrium (LA) due to AF have been studied extensively. A long PR interval and a wide P wave have a strong relationship with the LA remodeling of AF (11). Thus, microscopic changes in the ECG of patients with AF may appear in sinus rhythm. Moreover, these subtle changes will be clearer when the ECG of sinus rhythm is recorded close to the onset of AF.

We hypothesized that it would be possible to verify non-PeAF clinically in a shorter window period of less than 1 month. Therefore, this study was conducted to evaluate the possibility of risk stratification of non-PeAF based on a sinus rhythm ECG in window periods of 1, 2, and 4 weeks. In addition, we analyzed whether the diagnosis rate of non-PeAF increases with the increased proximity of AF and normal sinus rhythm (SR). Finally, to overcome the limitations of the black-box nature of AI, we checked the ECG area that the AI model focused on to predict non-PeAF using Grad-CAM, which is an attention technique.

## 2. Methods

### 2.1. Study population and ECG data

We enrolled adult patients (age 18–99 years) with sinus rhythm on a 12-lead standard ECG (10 seconds) that was recorded between 23 May 2017 and 2 September 2020 in Ewha Womans University, Mokdong Hospital, or Seoul Hospital, which are tertiary hospitals in Seoul, Republic of Korea. All ECGs were gathered from the emergency room, outpatient clinic, general ward, and intensive care unit and were obtained at a 500 Hz sample rate using Philips/General Electric (GE)/INFINITT ECG machines. All ECG data were recorded in an XML database. Ewha Womans University Mokdong Hospital Institutional Review Board approved (IRB File No. 2019-11-016-017) waiver of the requirement to obtain informed consent in accordance with 45 CFR 46.116.

### 2.2. ECG selection for analysis

**Figure 1:**
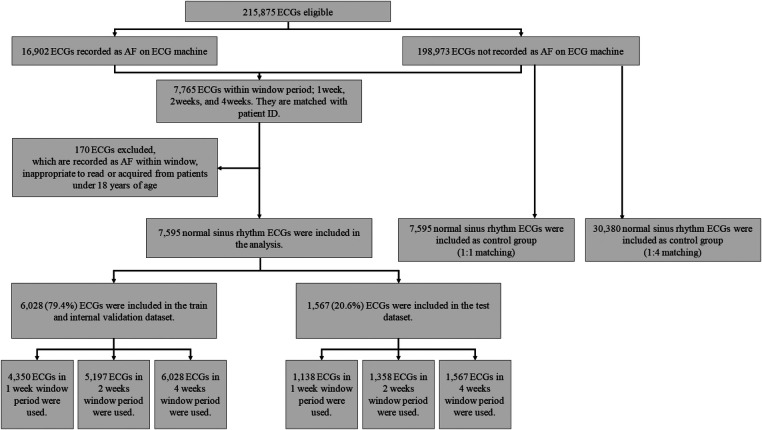
Patient flow diagram

**Figure 2:**
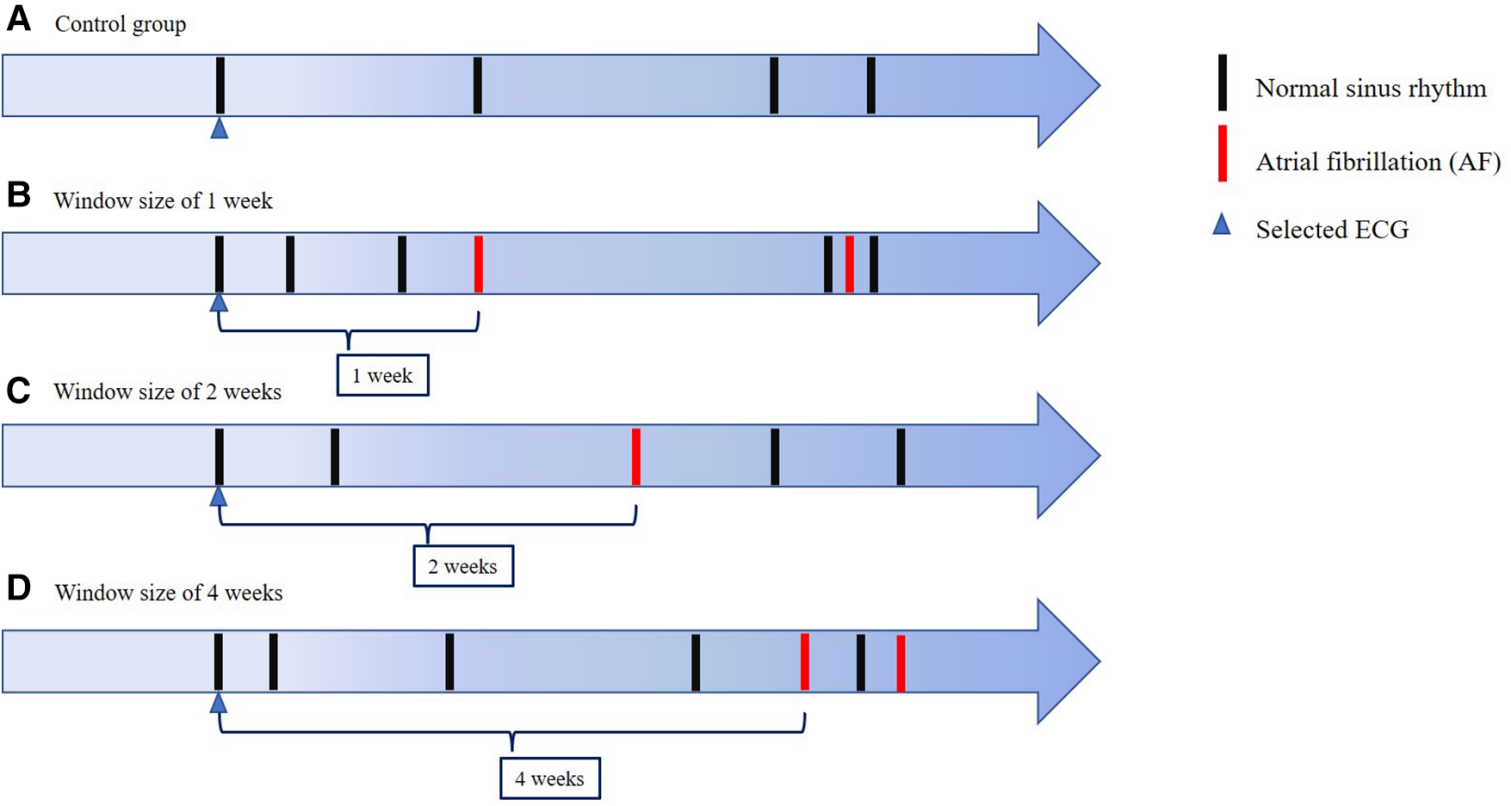
**ECG selection** The figure shows the schema of ECG selection for our CNN model and a control group. We used normal sinus rhythm ECGs obtained 1 week, 2 weeks and 4 weeks before AF detection prospectively. In the control group, we used normal sinus rhythm ECGs obtained from patients with no detected AF within the full observed period.

Among the 215,875 eligible ECGs that were identified, 16,902 and 198,973 were recorded as AF and SR, respectively (Figure 1). We selected 7,765 ECGs within a window period of interest – 1, 2, and 4 weeks from AF detection (Figure 2). The case sinus rhythm ECG (*n* = 7,595) were collected from patients who had AF ECGs in the window period. The control sinus rhythm ECG (*n* = 7,595 for 1:1 matching; *n* = 30,380 for 1:4 matching), for use as a control group, was defined as ECGs recorded from patients who had at least 3 ECGs recorded with SR in 1 year without a clinical diagnosis of AF. For training the algorithm, we collected control sinus rhythm ECGs according to 1:1 and 1:4 matching to consider the prevalence of AF compared to that of the case sinus rhythm.

### 2.3. Data preprocessing and attention technique

As described above, ECG data measured by an ECG machine manufactured by Philips, GE, or INFINITT were stored in XML format. As each stored format and the supported ECG have different leads, we parsed these data. In some cases, only two limb leads (Leads I and II) and six precordial leads were used. In such cases, Lead III was created based on Einthoven's law, and in the case of aVL, aVR, and aVF, data for these leads were derived using Goldberger's equations. Each data point was sampled at 500 Hz for 10 or 11 s. For ECGs with 11-second data, the data from the last 1 s were excluded and data from the first 10 s were used. In addition, as the input size of the model is 4,096, ECG data measuring 4,096 were constructed by truncating 600 data points and padding 148 zeros each at the beginning and the ending of the sequence which often contained noise. In this study, we adopted a previously proposed CNN model that comprises four ResBlocks (12) for non-PeAF. The model shows good performance in predicting diseases from ECG, as shown in the previous work, which showed F1-score of 0.870, precision of 1.000, sensitivity of 0.769 and specificity of 1.000. Moreover, we used Grad-CAM (13) to determine the points where the model paid attention to the ECG. The Grad-CAM presents the non-trainable attention of a CNN-based model that is calculated with the gradient inside the model by using a feature map output of the CNN which permits visualization of the point that the model focuses on.

### 2.4. Statistical analysis

Statistical analysis for the baseline characteristics with continuous and categorical variables was performed and we report their mean with standard deviation and numbers with percentages, respectively. Since there was no significant performance difference during iterative training, the average of the training results of the CNN-based model was adopted. After a final fitted model was obtained, the diagnostic performance was more formally analyzed. Measures of diagnostic performance included the area under the receiver operating characteristic curve (AUC), accuracy (i.e., a weighted average of sensitivity and specificity that indicates the percentage of patients whose labels were predicted correctly), sensitivity, specificity, and the F1 score (i.e., the harmonic mean of the sensitivity and positive predictive value). We used two-sided 95% CI to summarize the sample variability in the estimates. We used exact (Clopper–Pearson) CI to ensure a conservative approach to accuracy, sensitivity, and specificity. The CI for the AUC was estimated using the Sun and Su optimization of the Delong method by using the pROC package, whereas the CI for the F1 score was obtained using a bootstrap method with 2,000 replications. The whole procedure followed the standard approach in the machine learning field. All analyses were performed using IBM SPSS statistics, version 26.

## 3. Results

### 3.1. Baseline characteristics

In the analysis, we included the data of 94,780 patients, which comprised 215,875 ECGs; among them, there were 16,902 (7.83%) AF ECGs. After applying the exclusion criteria (*n* = 170), 7,595 case sinus rhythm ECGs with window periods of 1, 2, and 4 weeks were used for analysis as follows: 5,488, 6,555, and 7,595 ECGs, respectively. In addition, 20.6% of ECG data were used for testing whereas 79.4% of ECG data were used for training. In ECGs that were obtained with a 1-, 2-, or 4-week window period, the mean age of the participants was 70.60 ± 14.66, 69.58 ± 14.91, and 70.11 ± 14.42 years and 3,178 (57.9%), 3,546 (54.1%), and 4,063 (53.5%) were male, respectively. The mean age in the control group was 56.68 ± 17.23 years and 14,066 (46.3%) participants were male.

### 3.2. Early detection of non-PeAF based on the window period (1, 2, and 4 weeks)

**Figure 3:**
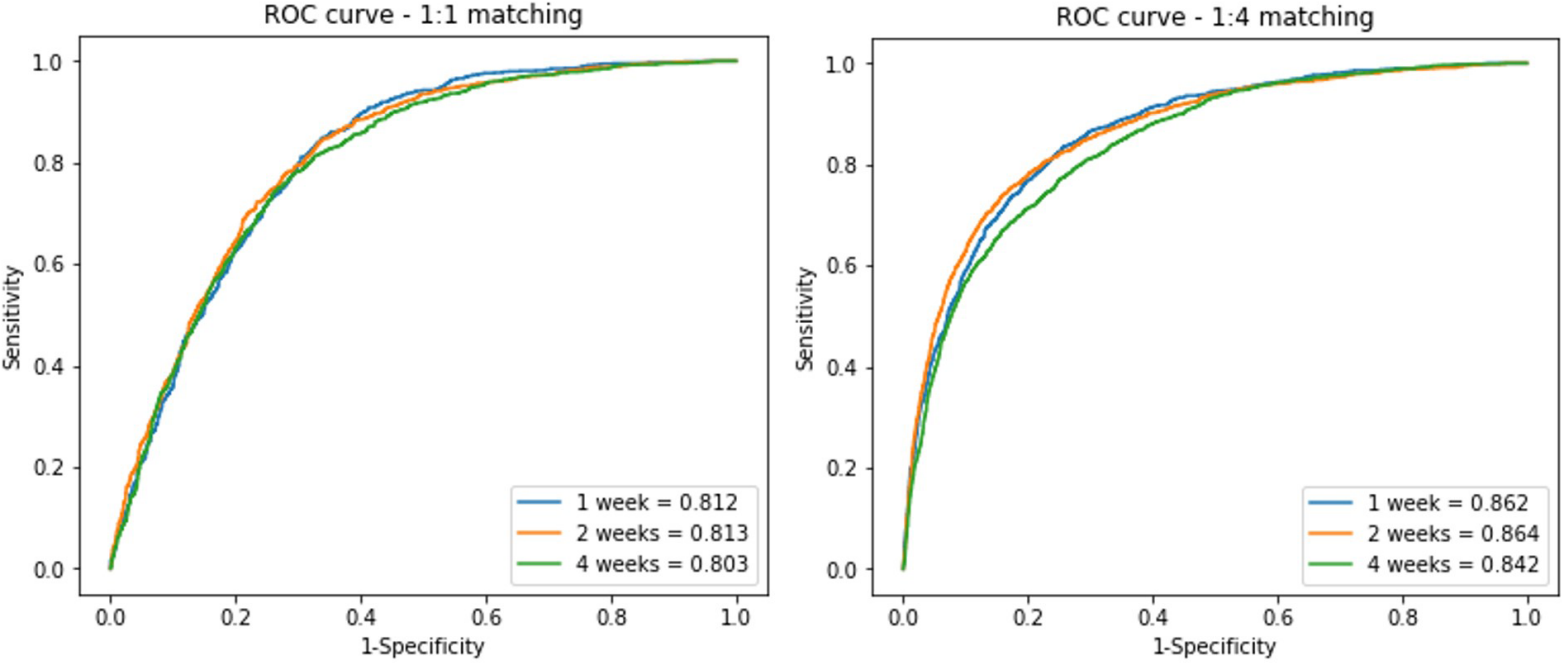
**ROC curves of the residual block model on the test data set** In the analysis, we divided the data into three window periods, which were defined with duration from NSR to PAF detection. 1:1 matched (**A**) and 1:4 matched (**B**) ECGs of control group included for analysis.

The algorithm was verified by internal validation using a test set and showed an AUC value of 0.812 (±0.001), 0.813 (±0.001), and 0.803 (±0.001); accuracy of 0.75, 0.75, and 0.74; and the same F1-score (0.74) for the 1-, 2-, and 4-week window periods, respectively. The AUC value of the model trained with the 1:4 matching data was 0.862 (±0.001), 0.864 (±0.001), and 0.842 (±0.001) for the 1-, 2-, and 4-week window periods, respectively (Figure 3).

A comparison of the training results using the 1:1 and 1:4 matching data showed no difference in the predictive power with regard to the AUC and F1 score. However, the sensitivity and specificity differed between the 1:1 and 1:4 matching data. In the 1:1 matching group, the sensitivity was high but specificity was low, whereas in the 1:4 matching group, the sensitivity was 48%–57%, but the specificity reached 93% (Table 1).

**Table 1:** **Model performance** Threshold for sensitivity, specificity, F1-score, and accuracy were set to 0.2 (± CI).

		AUC	F1 score	Accuracy	Specificity	Sensitivity (Recall)
1:1 matching	1 week	0.812(±0.001)	0.74(±0.009)	0.75(±0.009)	0.61(±0.013)	0.88(±0.009)
	2 weeks	0.813(±0.001)	0.74(±0.009)	0.75(±0.008)	0.63(±0.013)	0.86(±0.009)
	4 weeks	0.803(±0.001)	0.74(±0.009)	0.74(±0.009)	0.66(±0.013)	0.82(±0.010)
1:4 matching	1 week	0.862(±0.001)	0.84(±0.007)	0.84(±0.007)	0.91(±0.006)	0.57(±0.021)
	2 weeks	0.864(±0.001)	0.85(±0.008)	0.85(±0.007)	0.93(±0.006)	0.56(±0.022)
	4 weeks	0.842(±0.001)	0.83(±0.008)	0.84(±0.006)	0.93(±0.005)	0.48(±0.023)

### 3.3. ECG area to which the AI model mostly paid attention to predict AF using attention techniques

**Figure 4:**
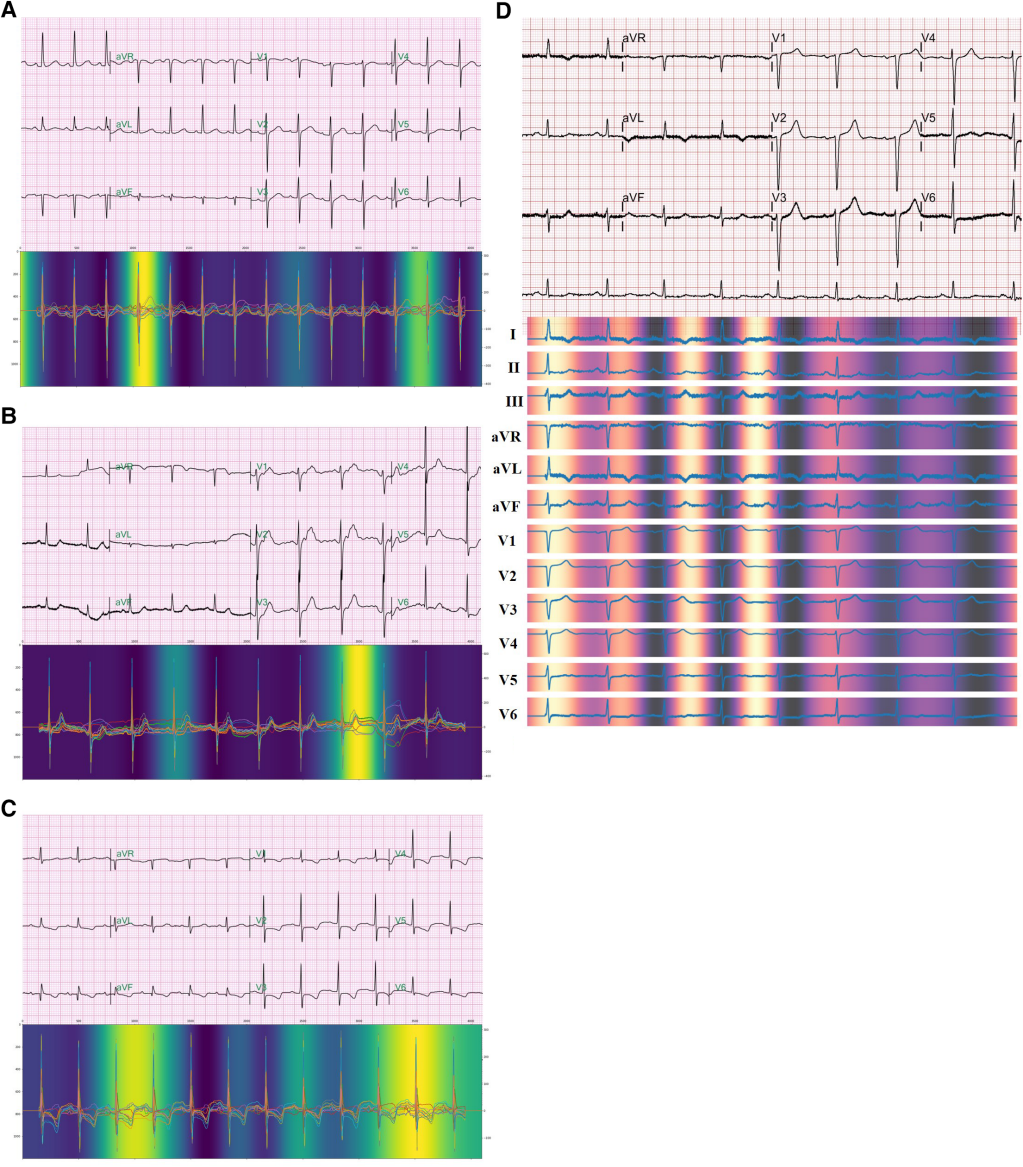
**Attention** The figure shows the points that the model paid attention to during the prediction occurrence of AF from normal sinus rhythm ECGs. Each ECG is selected within a window period of 1 week (**A**), 2 weeks (**B**), and 4 weeks (**C**). 12-lead ECG form of attention also shows highlight of a QRS wave in ECG selected within a window period of 1 week (**D**).

We used Grad-CAM to investigate the area to which the model paid attention to predict non-PeAF from SR ECGs. The Grad-CAM is the non-trainable attention of a CNN-based model, and is calculated with gradients by using a feature map output from the CNN, which visualizes the point that the model focuses on. SR ECGs within each window period were randomly selected respectively and confirmed through Grad-CAM. Therefore, one QRS wave area that was more brightly highlighted and one P-wave area that was relatively less bright were confirmed in a 1-week sample (Figure 4A). In a 2-week sample, a brightly highlighted T-wave area and a relatively less bright QRS-wave area were identified (Figure 4B). In a 4-week sample, one QRS wave area and one T wave area were brightly highlighted (Figure 4C). We confirmed that one QRS-wave area was highlighted even in the form of 12-lead ECG of attention, which was selected within a 1-week window period (Figure 4D).

## 4. Discussion

In this study, we developed and evaluated an AI model to predict AF from SR ECGs within a window period of 1, 2, and 4 weeks from AF, and analyzed the predictive performance according to the matching of the training/test sets. There are two interesting points found in this study; 1. Even with a short window period from an AF detection, the AI-based algorithm can predict non-PeAF within the window period. 2. The attention technique, Grad-CAM, showed the meaningful ECG area that is used for predicting non-PeAF from the sinus rhythm ECGs, and this finding could potentially play a pivotal role in the clinical implications of the AI prediction model.

### 4.1. Previous studies on the early detection of non-PeAF

Although early diagnosis of AF is clinically beneficial, the early diagnosis of PAF is very difficult. Approximately one-third of AF patients are asymptomatic, and it is difficult to conduct an ECG precisely during AF because symptom-onset is unpredictable (5–7). Therefore, if subtle traces of AF that appear on a sinus rhythm ECG are detected, patients who are at high risk for AF can be stratified early. Studies have investigated the possibility of an early diagnosis of AF by using a long PR interval and the P-wave amplitude (11, 14–16). Furthermore, an AF prediction model that used heartrate variability (HRV) in ECG data obtained for at least 5 min before AF documentation showed high specificity and sensitivity (17). However, the reproducibility of these studies was low, and the number of ECGs used in the analysis was small. Moreover, there exists a limitation in the clinical application of these methods because the predictive model was created from ECG information obtained over a relatively long period. In recent studies, approaches to AF prediction using AI algorithms are garnered the spotlight. Attia et al. (8) investigated a method for automatic prediction of AF based on a CNN model by using a short-term normal ECG signal, and showed that the closer the period (<30 days) between AF and sinus rhythm, the higher the diagnosis rate of AF. This finding provides evidence that the proximity of AF induces more subtle changes that can be seen in the sinus rhythm on ECG.

### 4.2. Clinical implications of this trial

In previous studies, higher model performance, sensitivity, and specificity were shown in an AI-based AF-prediction algorithm based on ECGs; however, these studies were limited by the fact that the ECG data which were used to predict PAF in most cases were either as short as 5 min or as long as 14 days. In addition, in many studies, these data were analyzed using an input signal of 300 s. We analyzed 12-lead ECG signals of the 10-second SR, which has an advantage of not requiring a long observation period, unlike that in previous studies (18–21). This trial verified potential of an AI algorithm on AF prediction with 12-lead SR ECG with Asian data, and predictive power in 1 week or 2 weeks was confirmed which provides the possibility of a prospective study followed closely.

A study used 12-lead ECGs and a 10-second signal (8), but the F1-score was rather low, and a major limitation of the study was that the window of interest of the occurrence of PAF was 31 days. We developed a model that can predict non-PeAF within a shorter window period (1, 2, and 4 weeks). Thus, the results of this study strengthens the possibility of predicting non-PeAF even with a 2-week window of interest and, in particular, the 1:4 matching group showed an AUC of 0.864, F1 score of 0.85, and high specificity that reached 93%. In the case of non-PeAF, which does not have a high fatality rate, our results suggest the clinical applicability of this model, given the high specificity. This study shows that the performance of the algorithm dose not decrease not only within 4 weeks of window period, but also within 1 or 2 weeks of window period.

We also considered age and sex information in our AI model by simply applying dense layers to this information and concatenating the result to that of our convolutional residual model. As such information was not available for every ECG; we used the mean value for the age and none for the sex. The performance gain, however, was not significant, that is, it was within ±1%. More specifically, for the 1:1 matching data, the performance slightly improved by up to about 1% when using the demographic information. On the contrary, for the 1:4 matching data, the performance slightly degraded by up to about 1% when using the demographic information. These findings indicate that sex or age does not serve as a confounding factor altering the performance of this AI model. As a result, the model can be applied universally, irrespective of sex or age. In future work, we will incorporate more demographic information to further enhance the performance of the AI model.

If the probability of AF occurrence is high according to the AI algorithm, then long-term Holter (wearable devices) monitoring in accordance with each window period can facilitate early diagnosis of non-PeAF. Thus, our study showed the usefulness of the novel model for the early diagnosis of non-PeAF. When it comes to palpitations, the estimated diagnostic yield for standard Holter monitoring (24–48 h) is 10%–15%, while ambulatory ECG (AECG) recordings spanning 1–4 weeks yield is around 70%–85%. However, for silent AF cases, the estimated diagnostic yield for standard Holter monitoring drops to 1%–5%, whereas that for AECG recordings spanning 1–4 weeks drops to 10%–15% (22). As AF often presents with vague symptoms or no symptoms at all, early screening for overall population requires excessive medical costs. Nevertheless, early detection of PAF is valuable as it can lead to improved patient outcomes by promptly identifying PAF before the progression of atrial cardiomyopathy. Therefore, this study aims to stratify the high-risk group expected to have non-PeAF, with can improve the diagnostic yield of AECG recording and enable cost-effective allocation of medical resources. Through the risk stratification, individuals at high risk for non-PeAF can be identified. By applying a wearable device for duration of 4 weeks or less, it is anticipated that valuable data such as the frequency and duration of AF can be obtained, along with the confirmation of diagnosis through a minimum recording of 30 s of AF on the ambulatory ECG (AECG).

### 4.3. Grad-CAM to unlock the “Black box”

Among the limitations of AI is the “Black box” nature, wherein the cause cannot be confirmed. There was no way to confirm whether the occurrence of AF was predicted according to any subtle changes in the ECG (23). Thus, the limitation that one cannot know whether specific and visibly distinguishable ECG changes were the basis for the prediction hinders the acceptance of clinicians because this detracts from complete trust or reliance on AI-based prediction models. To overcome this limitation, we used an attention technique. Although it was not possible to show an exact causal relationship through this approach, it was possible to at least infer whether the AI model was seeing the transformation of a clinically meaningful region. In this algorithm, we obtained evidence that the sinus rhythm ECG was used to predict AF through attention and discovered the possibility of potential clinical applications. Interestingly, when we checked attention, the points that the model paid attention to during prediction in all the window periods of 1, 2, and 4 weeks included one QRS point and one point between the T and P waves (Figures 4A–C). The QRS duration indicates conduction through the specialized cardiac conduction system and ventricular myocardium and is associated with cardiac structural and functional abnormalities (24). In previous studies, findings have indicated a relationship between the QRS wave and AF. After adjusting for potential AF risk factors, it was confirmed that a longer QRS duration was associated with a higher AF incidence rate in patients with LV dysfunction (25). Aeschbacher et al. (26) reported that QRS duration was an independent predictor of new-onset AF in women. The significance of this finding is that the attention of our AI prediction model shows that its prediction is based on sites that seem to have a relation when predicting the occurrence of AF from SR ECGs. Further research, however, is needed to determine how it differs from QRS waves at other sites.

The chronic problem with AI is that it has a “Black box” nature, as mentioned earlier. To solve this problem, attention mechanisms such as Grad-CAM have recently been extensively investigated. In this work, we trained the model, and we were able to determine which part of the ECG the model was focusing on through the Grad-CAM. However, as the ResBlock model employed in this study is based on 1D CNN, attention to each lead was not obtained. We expect to solve this by incorporating a trainable attention mechanism into the model.

This study attempts to present the possibility of a prospective study by verifying the previously suggested algorithm in Asian data and solving the problem of a black box for clinical application.

### 4.4. Limitations

One limitation of this study is its retrospective design and use of existing data; therefore, it is necessary to confirm the accuracy and clinical applicability of the AI predictive model through a prospective randomized study. The prediction by the AI algorithm is not strictly to be construed a prediction of future occurrence, but merely the detection of a subtle change in an ECG. For prediction of future occurrence of disease (non-PeAF), a prospective study should be needed for the AI algorithm. Additionally, there is a possibility that subjects classified within the control (SR) group, were misidentified, as instances of PAF might have been overlooked between the two time points of the 12-lead ECG. Furthermore, this study is limited by the absence of clinical data regarding the reasons why those ECGs were obtained and the corresponding clinical diagnosis of the subjects. We plan to undergo a prospective randomized control study by applying the developed algorithm in the near future.

## 5. Conclusion

The AI prediction algorithm showed the possibility of risk stratification for early detection of non-PeAF. Moreover, unlike previous studies, this study showed that a short window period is sufficient to detect non-PeAF.

## Data Availability

The raw data supporting the conclusions of this article will be made available by the authors, without undue reservation.
